# Complex trauma and perceived barriers to treatment among people accessing a supervised injecting facility

**DOI:** 10.1080/20008066.2025.2558241

**Published:** 2025-10-03

**Authors:** Ali Cheetham, Dan I. Lubman, Tina Lam, Elizabeth Grist, Anthony Barnett, Shalini Arunogiri, Suzanne Nielsen

**Affiliations:** a Monash Addiction Research Centre, Eastern Health Clinical School, Monash University Peninsula Campus, Frankston, Australia; b Turning Point, Eastern Health, Richmond, Australia; c National Centre for Youth Substance Use Research, School of Psychology, The University of Queensland, Brisbane, Australia

**Keywords:** People who inject drugs (PWID), supervised injecting facilities (SIFs), post-traumatic stress disorder (PTSD), complex PTSD (C-PTSD), barriers, TEPT complejo, TEPT, personas que se inyectan drogas (PID), centros de inyección supervisada (CIP), barreras al tratamiento

## Abstract

**Introduction:** People who inject drugs (PWID) have an elevated risk of trauma exposure, which can adversely affect health outcomes and create barriers to engaging with services. While high rates of post-traumatic stress disorder (PTSD) among PWID have been well-documented, less is known about the prevalence of complex PTSD (C-PTSD) in this cohort, or how symptom severity might relate to perceived barriers to alcohol and drug treatment.

**Methods:** Participants (*n* = 102; 74.5% male) were recruited from the Melbourne Supervised Injecting Room (MSIR) in Victoria, Australia, as part of a larger study examining the health needs of MSIR attendees. Participants completed a survey that included assessment of potentially traumatic life events (Life Events Checklist; LEC), past 30-day symptoms of PTSD/C-PTSD (International Trauma Questionnaire; ITQ), and perceived barriers to drug and alcohol treatment (Barriers to Treatment Inventory; BTI).

**Results:** Ninety-one participants (89.2%) had directly experienced at least one potentially traumatic event, with 24 participants (23.5%) endorsing events in 10 or more categories. Thirty participants (29.4%) met criteria for past 30-day PTSD; of these, 25 (83.3%) also met criteria for C-PTSD. C-PTSD symptom severity was associated with greater perceived barriers due to privacy concerns, lack of treatment availability, and time constraints, but lower barriers relating to perceived absence of a drug problem. PTSD symptom severity was associated with greater perceived barriers due to negative social support and lack of treatment availability. Females reported significantly greater trauma exposure and were more likely to meet PTSD/C-PTSD criteria than males.

**Discussion:** Cumulative trauma exposure and current C-PTSD symptoms were common among people attending a supervised injecting facility. Potentially, these PWID may recognise their need for drug treatment or services despite greater perceived structural and interpersonal barriers to treatment. Further research is needed to identify and address the unique barriers to accessing support services in these settings.

## HIGHLIGHTS

- High rates of lifetime trauma exposure and current PTSD and C-PTSD symptoms were found among people attending a supervised injecting facility, with females reporting significantly greater exposure and more severe symptoms than males.
- More severe symptoms of PTSD and C-PTSD were associated with greater perceived barriers to alcohol and drug treatment.
- Further research is needed to understand how experiences of complex trauma might impact help-seeking and use of support services among people who inject drugs.

## Abstract (es)

**Introducción:** Las personas que se inyectan drogas (PID) tienen un mayor riesgo de exposición a traumas, lo cual puede afectar negativamente sus resultados de salud y crear barreras para acceder a los servicios. Si bien las altas tasas de trastorno de estrés postraumático (TEPT) entre las PID están bien documentadas, se sabe menos sobre la prevalencia del TEPT complejo (TEPT-C) en esta cohorte, o sobre cómo la gravedad de los síntomas podría relacionarse con las barreras percibidas para el tratamiento del alcoholismo y las drogas.

**Métodos:** Los participantes (*n* = 102; 74,5% hombres) fueron reclutados en la Sala de Inyección Supervisada de Melbourne (MSIR por sus siglas en ingles) en Victoria, Australia, como parte de un estudio más amplio que examinó las necesidades de salud de las personas que asisten a la MSIR. Los participantes completaron una encuesta que incluyó la evaluación de eventos vitales potencialmente traumáticos (Lista de verificación de eventos vitales; LEC), síntomas de TEPT/TEPT-C de los últimos 30 días (Cuestionario internacional de trauma; ITQ) y barreras percibidas para el tratamiento de drogas y alcohol (Inventario de barreras para el tratamiento; BTI).

**Resultados:** Noventa y un participantes (89,2%) habían experimentado directamente al menos un evento potencialmente traumático, y 24 participantes (23,5%) identificaron eventos vitales en 10 o más categorías. Treinta participantes (29,4%) cumplieron los criterios para TEPT de los últimos 30 días; De estos, 25 (83,3%) también cumplían los criterios de TEPT-C. La gravedad de los síntomas de TEPT-C se asoció con mayores barreras percibidas para el tratamiento debido a preocupaciones sobre la privacidad, la falta de disponibilidad de tratamiento y las limitaciones de tiempo, pero con menores barreras relacionadas con la ausencia percibida de un problema de drogas. La gravedad de los síntomas de TEPT se asoció con mayores barreras percibidas debido al apoyo social negativo y la falta de disponibilidad de tratamiento. Las mujeres reportaron una exposición significativamente mayor al trauma y fueron más propensas a cumplir los criterios de síntomas de TEPT/TEPT-C que los hombres.

**Discusión:** La exposición acumulada al trauma y los síntomas actuales de TEPT-C fueron comunes entre las personas que asistían a un centro de inyección supervisado. Potencialmente, estas personas que se inyectan drogas podrían reconocer su necesidad de tratamiento o servicios para el alcohol y las drogas a pesar de percibir mayores barreras estructurales e interpersonales para el tratamiento. Se necesita más investigación para identificar y abordar las barreras específicas para acceder a los servicios de apoyo en estos entornos.

## 1. Introduction

Supervised injecting facilities (SIFs) provide a secure and hygienic environment in which people can inject drugs under the supervision of trained staff, as well as safer drug use education and emergency care in the event of an overdose. Also referred to as safe consumption sites (SCSs) and drug consumption rooms (DCRs), SIFs have been implemented as a public health response to drug-related harm across at least 17 countries since the 1980s (Colledge-Frisby et al., 2023). As facilities that support a marginalised population with a complex combination of health and social support needs (Kerman et al., 2020), most SIFs serve additional functions beyond the narrow range of services directly related to supervised consumption (EMCDDA, 2018). In Australia, two SIFs operate out of the two largest capital cities and are embedded within a public health framework that includes harm reduction and overdose prevention as well as linkages and referrals to a range of other health and social services, including alcohol and other drug and mental health treatment, medical and dental care, and housing and family violence support (EMCDDA, 2018). As many people who inject drugs (PWID) may have limited engagement with other mainstream services, SIFs can play an important role in reaching groups who lack material and social resources and may otherwise not seek help (Van Den Boom et al., 2021).

PWID have an elevated risk of exposure to trauma, which can create barriers to engaging with services, and highlights the importance of a trauma-informed approach to care for this population (i.e. one that recognises how trauma can affect people’s lives, and aims to provides services and environments that are safe, supportive, and sensitive to their needs) (SAMHSA, 2014). Over 90% of PWID report a history of traumatic life events (Goodhew et al., 2016; López-Castro et al., 2024; Peirce et al., 2012), including high rates of childhood trauma (Kennedy et al., 2020; Randhawa et al., 2018) physical and sexual assault (Goodhew et al., 2016; Kennedy et al., 2020; Peirce et al., 2012) and intimate partner violence (Kennedy et al., 2020; Peirce et al., 2012). Repeated exposures to trauma are common, with one prospective study finding 27% of PWID were re-exposed to a traumatic event each month over a 16-month follow up period (Peirce et al., 2012). Estimates of the prevalence of past 30-day post-traumatic stress disorder (PTSD) among PWID have ranged from 25% to 57.2% (Goodhew et al., 2016; Larance et al., 2015; López-Castro et al., 2024; Peirce et al., 2012), substantially higher than reported in samples of people with opioid use disorder (18.1%; Santo et al., 2022).

The prevalence of PTSD may be even higher among people who use SIFs. Compared to PWID who do not use these services or use them infrequently, people attending SIFs are more likely to report recent incarceration (Kennedy et al., 2019; Van Den Boom et al., 2021), homelessness (Scheim et al., 2021; Van Den Boom et al., 2021; Wood et al., 2005), selling drugs as a source of income (Scheim et al., 2021), and public injecting (Van Den Boom et al., 2021; Wood et al., 2005), increasing their risk of police intervention (Urbanik et al., 2022). In qualitative studies, people attending SIFs describe ‘routine’ experiences of violence and exploitation, witnessing overdose deaths, and frequent medical emergencies (Boyd et al., 2018; Fairbairn et al., 2008; Small et al., 2012), alongside complex trauma histories compounded by criminalisation, stigma, and social exclusion (Dertadian & Tomsen, 2022; Rickard & Hart, 2022). More frequent and intense experiences of violence have been reported by women (Ivsins et al., 2023), including victimisation by intimate partners as well as physical and sexual violence from strangers (Boyd et al., 2018; Fairbairn et al., 2008). One previous study has examined PTSD prevalence in this cohort, finding that 96% of people attending a SIF in Sydney, Australia, had experienced a traumatic event, with an average of 4.54 (±2.45) lifetime exposures and 3.04 (±2.50) exposures before the age of 16. One-third (36%) had symptoms of past 30-day PTSD, although only 12% had received a PTSD diagnosis from a professional (Goodhew et al., 2016).

A diagnosis of PTSD requires the presence of symptoms in three clusters: re-experiencing the event (e.g. through flashbacks or nightmares), avoidance of traumatic reminders, and heightened arousal (APA, 2013). However, more pervasive symptoms have been observed following prolonged or accumulated exposure to trauma, particularly in situations where escape is difficult or impossible. In 2018, complex PTSD (C-PTSD) was added to the World Health Organization’s International Classification of Diseases (ICD-11) (Reed et al., 2022), and is characterised by the core symptoms of PTSD in addition to symptoms reflecting ‘Disturbances in Self-Organisation’ (DSO). DSO symptoms fall into three clusters reflecting emotional dysregulation, negative self-concept (e.g. intense feelings of worthlessness or shame), and difficulties maintaining relationships (Cloitre et al., 2013). While strongly associated with childhood abuse (Cloitre et al., 2009), it has since been demonstrated that C-PTSD can also develop following exposure to prolonged and severe trauma in adulthood (Palic et al., 2016). Relative to PTSD, C-PTSD has been associated with greater levels of comorbidity and impairment, and lower quality of life (Cloitre et al., 2013).

While early and cumulative trauma have also been proposed to play a role in opioid use disorder (Garami et al., 2019), no studies to date have examined the prevalence of C-PTSD among PWID. Moreover, the impact of C-PTSD on service use among people with substance use disorder remains largely unexplored. In the broader mental health literature, PTSD has been associated with a lack of belief in the effectiveness of treatment, as well as concerns about stigma, shame, and rejection (Kantor et al., 2017). As prolonged or repeated exposure to interpersonal trauma can disrupt the capacity of survivors to trust others (Beaton & Thielking, 2020), people with C-PTSD may face additional barriers due to fear of the healthcare system, perceived mistreatment by staff, and a lack of appropriately skilled clinicians (de Boer et al., 2022; Williamson et al., 2021). People with experiences of complex trauma may also have additional needs, such as housing or legal support, that require them to navigate multiple services and agencies simultaneously (Salter et al., 2020). Understanding these trauma-related distinctions has particular relevance for SIFs. Unlike traditional healthcare settings where PWID may face drug-related stigma and discrimination and where drug use can even be an exclusion criteria for accessing services, SIFs can provide ‘low-threshold’ services that can be accessed without the cessation of drug use, and thereby can serve as important gateways to broader health and social support systems (Kerman et al., 2020; Van Den Boom et al., 2021). However, even within these supportive environments, the presence of C-PTSD versus PTSD may influence how individuals engage with services and staff (de Boer et al., 2022). For instance, the self-organisation difficulties characteristic of C-PTSD – including problems with emotional regulation, self-concept, and interpersonal relationships – may require different approaches to service delivery, such as longer relationship-building periods, trauma-informed communication strategies, and coordinated care planning that addresses the complex interplay between trauma symptoms and substance use. Further, symptoms reflecting disturbances in self-organisation could provide additional insight into why trauma exposure and PTSD among PWID has been associated with difficulties accessing health and social services (López-Castro et al., 2024; Peirce et al., 2012; Randhawa et al., 2018).

The current study aimed to provide insight into the prevalence and correlates of PTSD and C-PTSD symptoms among PWID through a secondary analysis of data from the second Review of the Medically Supervised Injecting Room (MSIR) in Victoria, Australia. Specifically, we aimed to establish the rate of trauma exposure, prevalence of current PTSD and C-PTSD symptoms, and the relationship between symptoms and perceived barriers to alcohol and drug treatment. Given evidence of gender differences in exposure to violence among people attending SIFs (Boyd et al., 2018; Fairbairn et al., 2008; Ivsins et al., 2023), we predicted that women would report a greater prevalence of trauma exposure and PTSD/C-PTSD than men. As no previous studies have examined C-PTSD among PWID, no hypotheses were specified regarding the prevalence or severity of C-PTSD symptoms. However, given the additional challenges posed by complex trauma, we expected C-PTSD symptom severity would be associated with a greater number of perceived treatment barriers.

## 2. Method

### 2.1. Participants

The MSIR was established in 2018 and provides supervised injecting services for over 6000 registered clients, as well as access to alcohol and other drug treatment, primary care, oral health, mental health support, referral to housing and homelessness services, and legal support. The current study was part of a larger mixed-methods project that aimed to explore the lived experiences of MSIR clients to describe their health, social, and welfare needs (including their level of trauma) and preferences for support/services offered at the MSIR.

### 2.2. Procedure

Participants were recruited directly from the MSIR between December 2021 and April 2022 with the assistance of on-site staff. Procedures varied due to COVID-19 restrictions on face-to-face contact, particularly during the earlier stages of recruitment. MSIR staff informed potential participants about the study and connected those who were interested to a member of the research team; where possible, a researcher was available on-site to conduct the survey in person. At other times contact details were collected via an expression of interest form, which MSIR staff then provided to the research team. Interruptions to on-site recruitment also occurred due to clinical incidents (i.e. an overdose in the recovery room requiring management). In order to minimise disruptions to the MSIR workflow, no data was collected from staff on how many participants were approached but declined to participate.

Participants were required to be at least 18 years of age, have attended the MSIR in the previous 6 months, and not be experiencing acute mental health symptoms that would preclude safe participation. The surveys took approximately 1 h and were undertaken either in person or over the phone (due to COVID restrictions). All participants provided informed verbal consent and were reimbursed with a $50 voucher. Ethics approval was obtained from the Monash University Human Research Ethics Committee (HREC 30754). Data were collected and managed using REDCap (Research Electronic Data Capture) (Harris et al., 2009, 2019) hosted on a secure server.

### 2.3. Measures

Domains assessed in the survey included demographics, past 30-day substance use, overdose and substance use treatment history (Manning et al., 2019), MSIR use frequency (participants who reported that more than 50% of their injections in the past month took place at the service were categorised as having ‘frequent’ MSIR use, in line with previously published research Van Den Boom et al., 2021), and a range of physical and mental health indicators (Ryan, 2023). Trauma exposure was measured using the Life Events Checklist (LEC-5; Gray et al., 2004), which includes 17 items measuring a range of potentially traumatic life events. Responses are rated on six nominal levels: ‘happened to me’, ‘witnessed it’, ‘learned about it’, ‘part of my job’ ‘not sure’, and ‘does not apply’. Responses were dichotomised into direct exposure (‘happened to me’) and all other categories, as this method of scoring has been shown to be more strongly predictive of PTSD (Weis et al., 2022), with the exception of sudden violent death/sudden unexpected death, where ‘witnessed it’ was categorised as direct exposure. The total number of categories in which events have been experienced was used as a measure of cumulative trauma exposure (Wilker et al., 2015). Separate scores were calculated for interpersonal trauma (physical assault, assault with a weapon, sexual assault, other unwanted or uncomfortable sexual experiences, combat or exposure to a war-zone, captivity, and serious injury/harm/death you caused to someone else; range 0–7) and non-interpersonal trauma (all other life event categories; range 0–10) (Ehring & Quack, 2010).

PTSD and C-PTSD symptoms were measured using the International Trauma Questionnaire (ITQ; Cloitre et al., 2018). The ITQ is an 18-item measure that includes six PTSD symptoms and six DSO symptoms, each followed by three items assessing past 30-day functional impairment. The six PTSD items assess the symptom clusters of re-experiencing the event, avoidance, and heightened arousal/threat. The six DSO items assess the symptom clusters of emotional dysregulation, negative self-concept, and difficulties in relationships. Scores for each symptom cluster were calculated by summing responses in each respective pair of items, with total PTSD and DSO scores calculated by summing responses to the three symptom clusters for each diagnostic category. A diagnosis of PTSD/C-PTSD requires a score ≥2 on one symptom in each of the three respective symptom clusters, plus a score ≥2 on at least one functional impairment indicator. If a person meets criteria for C-PTSD, they do not also receive a PTSD diagnosis.

Perceived barriers to alcohol and drug treatment were measured using the Barriers to Treatment Inventory (BTI; Rapp et al., 2006), which includes 25 items scored on a 5-point Likert scale ranging from 1 (‘strongly disagree’) to 5 (‘strongly agree’). BTI items encompass barriers in seven domains: Absence of a problem (6 items, e.g. *I do not think I have a problem with drugs*), Negative social support (5 items e.g. *my family will be embarrassed or ashamed if I go to treatment*), Fear of treatment (4 items e.g. *I have had a bad experience with treatment*), Privacy concerns (3 items e.g. *I do not like to talk about my personal life with other people*), Time conflict (e.g. *it will be hard for me to find a treatment program that fits my schedule*), Treatment availability (4 items e.g. *I do not know where to go for treatment*), and Admission difficulty (3 items e.g. *I have to go through too many steps to get into treatment*). Higher mean scores for each subscale indicated greater perceived barriers.

### 2.4. Data analysis

IBM Statistical Package for Social Sciences (SPSS) version 24.0 was used to analyse the data (IBM Statistics, 2013). Missing data on demographic variables and the BTI was low (<5%), however there was a moderate-to-high rate of participant-level missing data on the ITQ (17.6%) and LEC (4.9%). For the remaining participants, missing data at the item level ranged between 0–5.3% for the ITQ and 0–7.1% for the LEC. In addition, one item from the C-PTSD symptom cluster ‘disturbances in relationships’ was accidentally omitted from the Redcap survey. Where possible, this was imputed using the method described by Panayi and colleagues (i.e. if one of the two symptom cluster scores were missing, the score on the other item was imputed; if one functional impact score was missing, the mean of the other two scores was imputed Panayi et al., 2022).

Chronbach alpha values were acceptable for the ITQ (PTSD *α* = 0.85, C-PTSD *α* = 0.91) and BTI subscales (*α* = 0.65–0.80). Descriptive data are presented as percentages, medians, and means (SD). Gender differences in trauma exposure and PTSD symptoms were examined using chi-squared analyses. Relationships between PTSD and DSO scores and sociodemographic variables were explored using independent samples t-tests and Pearson correlation. Pearson correlation was used to examine relationships between PTSD and DSO symptom clusters and perceived barriers to treatment at the subscale level. Additional analyses were undertaken to compare participants who did and did not complete the ITQ on key variables.

## 3. Results

### 3.1. Demographics

One-hundred and two participants completed the survey. Demographic and substance use characteristics (for the full sample, and separately by gender) are presented in Table 1. Compared to males, females were less likely to report paid employment and more likely to report chronic medical conditions. There were no other differences in demographic or substance use variables between genders.

**Table 1.** Demographic characteristics, substance use, and International Trauma Questionnaire (ITQ) results by gender.

	Total (*n* = 102)	Male (*n* = 77)	Female (*n* = 25)	χ*^2^*(1)
Demographic and substance use characteristics				* *
Age (mean, SD)	44.2 (8.6)	44.8 (8.3)	42.2 (9.5)	*ns*
Gender (% male)	74.5	–	–	*ns*
Aboriginal or Torres Strait Islander (%)	21.6	22.1	20.0	*ns*
Usually in paid employment (past 12 months, %)	14.7	19.5	0	*χ^2^*(1) = 5.710, *p* = .017
Housing problems/homelessness (past 90 days, %)	30.4	28.6	36.0	*ns*
Current legal issue (%)	47.1	48.0	44.0	*ns*
Chronic medical condition (%)	43.1	32.5	76.0	*χ^2^*(1) = 14.581, *p* < .001
Frequent MSIR use ^a^	66.7	64.9	72.0	*ns*
Duration of heroin use in years (median, range)	23.9 (9.5)	24.1 (9.2)	23.3 (10.3)	*ns*
Ever sought treatment for heroin use (%)	87.3	85.7	92.0	*ns*
No. days heroin used (past 30 days) ^b^	20.9 (10.2)	20.8 (10.3)	21.2(10.1)	*ns*
Daily heroin use (past 30 days, %)	35.3	35.1	36.0	*ns*
Accidentally overdosed on opioids (lifetime, %)	71.6	72.7	68.0	*ns*
Recent overdose (past 12 months; %)	32.4	33.8	28.0	*ns*
ITQ				* *
Past 30-day PTSD (%)^c^	36.6	17.3	40.0	*χ*^2^(1) = 5.142(1), *p* = .023
PTSD score (mean, SD)	11.02 (7.29)	9.28 (7.03)	15.77 (5.84)	*t*(80) *= *−3.865, *p *= <.001
DSO score (mean, SD)	12.71 (7.88)	11.16 (8.08)	16.73 (5.72)	*t*(77) *= *−2.954, *p* = .004

^a^ > 50% of their injections in the past month took place at the service (Van Den Boom et al., 2021); ^b^ Log transformed prior to analysis; 9 participants had used heroin but had missing data on the number of days; ^c^ *n* = 82 (male = 61, female = 21).

### 3.2. Prevalence of trauma exposure

Ninety-one participants (89.2%) reported experiencing at least one potentially traumatic life event. Nine participants (8.8%) had not experienced any potentially traumatic events (*n* = 6), or did not consider the events they had experienced to be distressing (*n* = 3). Five participants (4.9%) did not complete the LEC or had missing data on >30% of life event categories; data from these participants was not included in analyses examining the total number of life event categories experienced.

Table 2 presents the number and percentage of participants who endorsed each item on the LEC. The mean number of categories endorsed was 6.56 (SD = 4.16, range 0–15; median 6.00), with 89 participants (87.3%) endorsing more than one category, and 24 participants (23.5%) endorsing 10 or more categories. Females reported events in a greater number of categories (median 8.00; mean = 8.20, SD = 3.88) compared to males (median 5.00; mean = 6.00, SD = 4.12; *t*(95) = −2.348, *p* = .021), with significantly greater exposure to interpersonal trauma (mean = 4.08, SD = 1.50 vs mean = 2.44, SD = 1.90; *t*(95) = −3.924, *p* < .001), but not non-interpersonal trauma (mean = 4.12, SD = 2.79 vs mean = 3.51, SD = 2.56; *t*(95) = −0.996, *p* = .322).

**Table 2.** Number of Life Events Checklist (LEC) categories endorsed by gender.

	No. reporting direct exposure (*n*, %)					
Life event category	Total (*n* = 102)		Male (*n* = 77)		Female (*n* = 25)	
Physical assault	77	78.6%	54	74.0%	23	92.0%
Assault with a weapon	67	67.7%	45	60.8%	22	88.0%
Transportation accident	56	58.3%	42	59.2%	14	56.0%
Life threatening illness or injury	46	47.4%	34	47.2%	12	48.0%
Sexual assault	43	44.3%	24	33.3%	19	76.0%
Any other very stressful event or experience	43	45.7%	26	37.7%	17	68.0%
Any other unwanted sexual experience	41	41.8%	23	31.5%	18	72.0%
Serious accident at work or home	40	40.8%	31	42.5%	9	36.0%
Sudden accidental death	36	34.0%	26	36.1%	10	40.0%
Sudden violent death	34	36.0%	25	35.2%	9	39.1%
Fire or explosion	32	33.0%	22	29.7%	10	43.5%
Captivity	30	30.6%	17	23.3%	13	52.0%
Natural disaster	29	29.3%	20	27.0%	9	36.0%
Severe human suffering	26	28.3%	17	23.6%	9	39.1%
Exposure to toxic substances	18	18.4%	14	19.2%	4	16.0%
Severe harm or death you caused	16	17.0%	12	17.4%	4	16.0%
Combat or exposure to a war zone	10	10.4%	7	9.9%	3	12.0%

Seventy-five participants (73.5%) completed the ITQ with reference to a potentially traumatic event (of these, two did not disclose the nature of the event due to distress). Of those who did not complete the ITQ, nine participants (8.8%) had not experienced any serious life events (*n* = 6) or did not consider the events they had experienced to be distressing (*n* = 3); eighteen participants (17.6%) were coded as missing data. Sixty-six participants provided a brief description of the event they considered the most distressing. Interpersonal violence of any kind featured in 24 descriptions, with the most common specific events including sudden violent or unexpected death (*n* = 16), sexual assault (*n* = 11), transportation accidents (*n* = 10), and ‘other’ experiences (*n* = 11; e.g. loss of custody of a child or children, experiences in the court system, mental health episodes, threats to life, and child abuse). Of the events described, 23.6% occurred within the last 12 months, 13.2% occurred 1–5 years ago, 26.5% occurred 5–20 years ago, and 36.8% occurred more than 20 years ago.

### 3.3. Prevalence and correlates of PTSD and C-PTSD

Omission of the C-PTSD item did not alter estimates of C-PTSD (i.e. all participants who scored ≥2 on the other C-PTSD symptom pairs and functional impairment items scored ≥2 on the remaining ‘disturbances in relationships’ item; no participants with a score of ≤2 on the remaining ‘disturbances in relationships’ item would have otherwise met criteria for C-PTSD). However, two participants had missing data for all three PTSD functional impairment items, precluding categorisation in the PTSD, C-PTSD, or no disorder groups, while DSO total scores were not calculated for three participants who had missing data on both items in one or more C-PTSD symptom clusters. PTSD/C-PTSD was coded as absent for the nine participants who did not complete the ITQ because they had not experienced any potentially traumatic events or consider the events they had experienced to be distressing.

Thirty participants (29.4%) met criteria for past 30-day PTSD, 52 (50.9%) did not meet criteria, and 20 (19.6%) had missing data. Participants who met criteria for PTSD had an average total PTSD score of 17.63 (SD = 4.24). Twenty-five participants (83.3%) with PTSD also met criteria for C-PTSD, with an average total DSO score of 18.36 (SD = 4.07). Five participants (4.9%) were coded as PTSD only. Compared to males, females were significantly more likely to meet criteria for past 30-day PTSD/C-PTSD, and reported higher total PTSD and DSO scores (Table 1). There were no differences in PTSD or DSO scores between participants grouped on the basis of any substance use variables (MSIR use frequency, past 30-day heroin use, daily heroin use, lifetime or past 12-month overdose; all *p* values > .485).

### 3.4. Barriers to treatment

Pearson correlation coefficients for ITQ symptom clusters and BTI subscales are presented in Table 3. Total PTSD scores were positively correlated with negative social support (*r*(80) = .249, *p* = .025) and treatment availability (*r*(80) = .242, *p* = .029). Total DSO scores were negatively correlated with absence of a problem (e.g. did not think their drug use was a problem) (*r*(77) = −.311, *p* = .005), and positively correlated with privacy concerns (*r*(77) = .276, *p* *=* .014), time (*r*(77) = .262, *p* = .020), treatment availability (*r*(77) = .399, *p *= <.001). The correlation between total DSO scores and fear of treatment did not reach the threshold for statistical significance (*r*(77) = .214, *p* *=* .058). There were no gender differences on any BTI subscales (all *p* values > .189).

**Table 3.** Pearson correlation coefficients indicating the relationship between International Trauma Questionnaire (ITQ) symptom clusters and Barrier to Treatment Inventory (BTI) subscales.

	PTSD symptoms			DSO symptoms		
Barrier subscale	Re-experiencing	Avoidance	Sense of threat	Affective dysregulation	Negative self-concept	Disturbances in relationship
Absence of problem	−.174	−.249*	−.019	−.176	−.322**	−.357**
Negative social support	.257*	.203	.184	.142	.173	.167
Fear of treatment	.149	.167	.139	.207	.206	.181
Privacy concerns	.089	.099	−.025	.257*	.288**	.223*
Time	.153	.149	.150	.282*	.232*	.214
Treatment availability	.244*	.090	.284*	.416***	.388***	.305**
Administrative difficulties	.095	.080	.070	.102	.159	.103

*Significant at <0.05; **Significant at <0.01; **Significant at <0.001.

### 3.5. Differences between ITQ responders and non-responders

Of the 18 participants with missing ITQ data, 3 (16.7%) also did not complete the LEQ. For the remaining 15 non-responders, the total number of potentially traumatic events experienced (M = 6.73, SD = 4.28) was similar to responders (M = 6.39, SD = 4.02; *t*(95) = 0.300, *p* = .764). Responders and non-responders did not differ in regard to demographic characteristics, including gender, or perceived barriers to treatment (all *p* values > .143). Seventeen participants with missing ITQ data completed all subsequent survey questions, with a review of interviewer notes identifying three participants who declined to answer questions about trauma due to distress.

## 4. Discussion

High rates of trauma exposure and past 30-day symptoms of PTSD were found among people attending a SIF in Melbourne, Australia. Our findings corroborate descriptions of trauma among SIF clients, most notably frequent experiences of violence and high rates of cumulative trauma exposure (Dertadian & Tomsen, 2022; Rickard & Hart, 2022; Small et al., 2012), with disproportionate risk among women (Ivsins et al., 2023). The prevalence of PTSD in the current study (29.4%) is comparable to a previous study of people attending a SIF (36%; Goodhew et al., 2016). The majority of participants (83.3%) who met criteria for PTSD in the current study also met criteria for C-PTSD, aligning with the endorsement of multiple potentially traumatic events on the LEC. While early and cumulative trauma have been proposed to have relevance to opioid use disorder (Garami et al., 2019), to our knowledge this is the first study to examine the prevalence of C-PTSD among people attending SIFs or PWID more broadly.

As expected, DSO symptom severity was correlated with a greater number of perceived barriers to treatment. Participants with more severe PTSD symptoms were less likely to engage with services due to negative reactions from others and lack of treatment availability, while participants with more severe DSO symptoms were less likely to engage with services due privacy concerns, lack of time, and lack of treatment availability. More severe DSO symptoms were also associated with *lower* barriers relating to absence of a problem, suggesting that PWID with experiences of complex trauma may recognise their need for care despite perceiving a greater number of other barriers to receiving treatment. These findings are consistent with evidence from the broader literature examining barriers among people with PTSD (Kantor et al., 2017), as well as recent research that has emphasised the importance of addressing both intrapersonal and structural barriers in populations with complex trauma exposure (de Boer et al., 2022).

The correlations between ITQ symptom clusters and BTI subscales suggest that establishing trust and psychological safety may be particularly important for PWID with complex trauma. Concerns about privacy were associated with the DSO symptom subscale of negative self-concept, and to a lesser extent affective dysregulation and disturbances in relationships. A reluctance to disclose personal information is likely to be compounded among people attending SIFs through prior experiences with the police and criminal justice system, as well as stigma and discrimination in health services (Urbanik et al., 2022). Conversely, trust has been recognised as a valuable resource for the delivery of effective health care for PWID (Treloar et al., 2016), and ongoing relationships with staff can play an important role in facilitating open discussions about life circumstances, drug consumption, and health needs (Rance & Fraser, 2011). In addition to providing SIF staff with ongoing training in the delivery of trauma-informed care, ensuring continuity of staffing in order to build trusting relations may help to address the barriers to treatment experienced by PWID with trauma experiences. Implementing these recommendations would necessitate structural supports including stable funding for core staff positions, policy frameworks that mandate and support trauma-informed care training, and inter-agency agreements that facilitate coordinated service delivery for individuals with complex trauma histories. Further, despite established evidence for broader integrated care approaches for co-occurring mental health and substance use disorders, including collaborative care models and co-located services, structural barriers such as funding silos and workforce shortages continue to impede widespread or sustainable implementation of these evidence-based practices for PWID (Cheetham et al., 2023).

More frequent interactions with service systems could contribute to perceived structural barriers among people with complex trauma. Associations with affective dysregulation and negative self-concept are consistent with research that has found a lack of support for people with experiences of complex trauma can escalate their needs until they present in crisis (Salter et al., 2020). More generally, the greater likelihood that PWID with complex trauma will need to navigate multiple services and agencies simultaneously is likely to make engagement with any single service more challenging (Salter et al., 2020). Intensive referral and case-management in SIFs can increase the uptake of drug treatment for people with psychiatric comorbidity (Kimber et al., 2008), however our findings also highlight the need for services to incorporate principles of trauma-informed care (SAMHSA, 2014). Drug treatment services that do not adequately screen for trauma (Harvey et al., 2023) or respond in a manner that is overly prescriptive or inflexible, without considering potential relationships between trauma and drug use (Breckenridge et al., 2012), may heighten perceived structural barriers.

We found no gender differences in perceived barriers despite greater trauma exposure and symptom severity among women, who also reported lower rates of paid employment and higher rates of chronic medical conditions compared to men. Previous studies have found the BTI to be invariant across gender (Rapp et al., 2006), however a recent study including additional items that disproportionately impact women found greater barriers due to family responsibilities, relational factors, and mental health (Agterberg et al., 2020). Qualitative studies have found a strong preference for women-only supervised consumption services among PWID (Ivsins et al., 2023), potentially due to patterns of violence towards women that extend into mixed-gender SIF settings (Boyd et al., 2018). As the protective effects of SIFs on exposure to violence do not appear to be experienced equally by males and females (Kennedy et al., 2020), there may be additional barriers that relate to the unique needs of women in these settings. Recent Australian research has found that a perceived lack of treatment flexibility is a more significant barrier to opioid dependence treatment among women than men (Hall et al., 2023). In addition, women report higher levels of perceived stigma for seeking substance use disorder treatment (Agterberg et al., 2020), are more vulnerable to the disruptive effects of housing instability, healthcare access, and financial barriers (Chavez et al., 2025), and can experience multiple compounding stressors that intensify challenges in accessing treatment (Spector et al., 2021).

We found no association between PTSD or DSO scores and measures of opioid use, treatment history, or overdose. Several theories have been proposed to explain the high co-morbidity of PTSD and substance use disorders, including that people use substances to alleviate symptoms of trauma, and, conversely, that substance use increases the risk of being exposed to traumatic events (Brady et al., 2021; Garami et al., 2019). However, assessment of opioid use was not a primary aim of the larger MSIR project, and more granular assessment would be needed to account for its highly complex relationship with trauma. The lack of associations could have been due to a lack of statistical power or the use of largely categorical outcome variables, both of which could be explored in future research that takes a more comprehensive substance use history.

The findings of the current study provide information about experiences of trauma and perceived barriers to treatment in a population where this evidence is currently lacking. However, our study has a number of limitations, most notably the small sample size and high rate of missing data on the ITQ. The pattern of missing data, where participants skipped only the trauma questions, along with evidence that some were too distressed to discuss traumatic events in detail, suggests that data may not have been obtained from participants with more severe symptoms. While there were no differences between participants who did and did not complete the ITQ in regard to trauma exposure or perceived barriers to treatment, this remains a likely source of bias that limits interpretation of results regarding the relationship between trauma experiences and perceived barriers to treatment. Participants with more severe symptoms may also have declined to participate in the project, or may not have been approached by MSIR staff (or deemed ineligible) if experiencing acute distress at the time of recruitment. As such, the current results may not reflect the experiences of those who face the greatest challenges in accessing and remaining in treatment due to trauma. More broadly, while missingness did not appear to be associated with participant demographics, the small sample size and convenience sampling approach limits the extent to which our results can be generalised to the wider population of people who attend SIFs. Given these limitations, further research among people recruited from SIFs is needed to confirm the associations found in the current study. Greater consideration of this population is warranted given that studies have repeatedly found PWID who use SIFs differ significantly from those who do not in regard to homelessness, incarceration, and other measures of disadvantage that are likely to be meaningfully related to trauma (Kennedy et al., 2019; Scheim et al., 2021; Van Den Boom et al., 2021; Wood et al., 2005).

This study employed an ICD-11 conceptualisation of PTSD, limiting comparisons to broader literature (particular in the North American setting) utilising a DSM-5 framework with a broader definition of symptom profiles, including negative alterations in mood, arousal and cognition. Additional limitations include a lack of assessment of trauma in childhood, as research suggests that the contribution of cumulative trauma in adulthood to symptom complexity is determined by the presence of cumulative childhood trauma (Cloitre et al., 2009). Measuring trauma exposure by types rather than discrete events is also likely to underestimate the actual number of traumatic events experienced, although this method has been shown to be reliable (Wilker et al., 2015) and may more strongly predict PTSD symptoms in settings with more opportunities for trauma exposure (Rasmussen et al., 2020). As the cross-sectional nature of the data limits conclusions about causal relationships, a more comprehensive, longitudinal assessment of substance use (including poly-substance use) may also help clarify relationships between symptoms of PTSD and C-PTSD and outcomes, including overdose. Finally, the small number of women precluded further analyses on gender differences in the relationship between trauma exposure and barriers to treatment. While the ratio of males to females was consistent with the gender balance reported in previous studies of SIFs (Goodhew et al., 2016; Van Den Boom et al., 2021), dedicated research is needed to better understand barriers among women who use these services (Boyd et al., 2018).

## 5. Conclusion

SIFs are established primarily as a physical location to inject drugs, with the range of ancillary services provided varying considerably across countries and settings. However, they provide a unique support network for a marginalised population, with opportunities to maintain engagement among hard-to-reach groups who may lack the material and social resources to engage with services. For facilities such as the MSIR that have been established with the aim of providing a gateway to more effective health and social assistance, our findings support that mental health wrap-around services continue to be considered intrinsic to SIF operations. While further research is needed to understand relationships between C-PTSD and barriers to treatment, identifying C-PTSD among SIF attendees could inform the development of tailored interventions and staff training programmes that better support this population's unique needs. Rather than simply recognising trauma exposure, distinguishing between PTSD and C-PTSD symptom profiles and highlighting impact on relational dynamics could guide practical decisions about service intensity, referral pathways, and the types of wrap-around supports most likely to facilitate engagement with both addiction treatment and trauma recovery services.

It is also important to consider that for a highly marginalised population with significant exposure to trauma, building a sense of trust and meeting basic needs (such as food and shelter) may better position them to feel able to engage in treatment. In addition, all SIF staff should be provided with ongoing training informed by principles of trauma-informed care, and organisations should work to create supportive environments that prioritise staff wellbeing, recognising that trauma-informed care also includes addressing vicarious trauma and burnout. Future research may benefit by consulting communities of PWID accessing SIFs, and staff working within them, to identify and address the unique barriers to accessing various SIF support services for individuals with trauma or psychological distress.

## Author contribution

AC: Conceptualisation, Methodology, Data curation, Formal analysis, Writing – Original Draft, Writing – Review & Editing. DL: Writing – Review & Editing, Supervision, Funding acquisition. TL: Data curation, Writing – Review & Editing, Funding acquisition. EG: Conceptualisation, Writing – Original Draft, Writing – Review & Editing. AB: Conceptualisation, Methodology, Writing – Review & Editing, Funding acquisition; SA: Conceptualisation, Methodology, Writing – Original Draft, Writing – Review & Editing, Supervision, Funding acquisition. SN: Conceptualisation, Methodology, Writing – Original Draft, Writing – Review & Editing, Supervision, Funding acquisition.

## Data, material, and/or code availability

The participants of this study did not give written consent for their data to be shared publicly, so due to the sensitive nature of the research supporting data is not available.

## References

[CIT0001] Agterberg, S., Schubert, N., Overington, L., & Corace, K. (2020). Treatment barriers among individuals with co-occurring substance use and mental health problems: Examining gender differences. *Journal of Substance Abuse Treatment*, *112*, 29–35. 10.1016/j.jsat.2020.01.005 32199543

[CIT0002] American Psychiatric Association (APA). (2013). *Diagnostic and statistical manual of mental disorders (DSM 5)*.

[CIT0003] Beaton, J., & Thielking, M. (2020). Chronic mistrust and complex trauma: Australian psychologists’ perspectives on the treatment of young women with a history of childhood maltreatment. *Australian Psychologist*, *55*(3), 230–243. 10.1111/ap.12430

[CIT0004] Boyd, J., Collins, A. B., Mayer, S., Maher, L., Kerr, T., & McNeil, R. (2018). Gendered violence and overdose prevention sites: A rapid ethnographic study during an overdose epidemic in Vancouver, Canada. *Addiction*, *113*(12), 2261–2270. 10.1111/add.14417 30211453 PMC6400212

[CIT0005] Brady, K. T., McCauley, J. L., & Back, S. E. (2021). The comorbidity of post-traumatic stress disorder (PTSD) and substance use disorders. *Textbook of Addiction Treatment: International Perspectives*, 1327–1339. 10.1007/978-3-030-36391-8_93

[CIT0006] Breckenridge, J., Salter, M., & Shaw, E. (2012). Use and abuse: Understanding the intersections of childhood abuse, alcohol and drug use and mental health. *Mental Health and Substance use*, *5*(4), 314–327. 10.1080/17523281.2012.703224

[CIT0007] Chavez, C. L. J., Peltier, M. R., & McKee, S. A. (2025). Sex differences in the impact of social determinants of health on substance use disorder treatment outcomes. *Biology of Sex Differences*, *16*(1), 56. 10.1186/s13293-025-00734-3 40696464 PMC12281950

[CIT0008] Cheetham, A., Arunogiri, S., & Lubman, D. (2023). Integrated care–panacea or white elephant? A review of integrated care approaches in Australia over the past two decades. *Advances in Dual Diagnosis*, *16*(1), 3–16. 10.1108/ADD-10-2022-0026

[CIT0009] Cloitre, M., Garvert, D. W., Brewin, C. R., Bryant, R. A., & Maercker, A. (2013). Evidence for proposed ICD-11 PTSD and complex PTSD: A latent profile analysis. *European Journal of Psychotraumatology*, *4*(1), 20706. 10.3402/ejpt.v4i0.20706 PMC3656217 23687563

[CIT0010] Cloitre, M., Shevlin, M., Brewin, C. R., Bisson, J. I., Roberts, N. P., Maercker, A., Karatzias, T., & Hyland, P. (2018). The International Trauma Questionnaire: Development of a self-report measure of ICD-11 PTSD and complex PTSD. *Acta Psychiatrica Scandinavica*, *138*(6), 536–546. 10.1111/acps.12956 30178492

[CIT0011] Cloitre, M., Stolbach, B. C., Herman, J. L., Kolk, B. v. d., Pynoos, R., Wang, J., & Petkova, E. (2009). A developmental approach to complex PTSD: Childhood and adult cumulative trauma as predictors of symptom complexity. *Journal of Traumatic Stress*, *22*(5), 399–408. 10.1002/jts.20444 19795402

[CIT0012] Colledge-Frisby, S., Ottaviano, S., Webb, P., Grebely, J., Wheeler, A., Cunningham, E. B., Hajarizadeh, B., Leung, J., Peacock, A., Vickerman, P., Farrell, M., Dore, G. J., Hickman, M., & Degenhardt, L. (2023). Global coverage of interventions to prevent and manage drug-related harms among people who inject drugs: A systematic review. *The Lancet Global Health*, *11*(5), e673–e683. 10.1016/S2214-109X(23)00058-X 36996860 PMC12765503

[CIT0013] de Boer, K., Arnold, C., Mackelprang, J. L., & Nedeljkovic, M. (2022). Barriers and facilitators to treatment seeking and engagement amongst women with complex trauma histories. *Health & Social Care in the Community*, *30*(6), e4303–e4310. 10.1111/hsc.13823 35545923 PMC10084282

[CIT0014] Dertadian, G., & Tomsen, S. (2022). The experience of safety, harassment and social exclusion among male clients of Sydney's Medically Supervised Injecting Centre. *International Journal for Crime, Justice and Social Democracy*, *11*(4), 13–24.

[CIT0015] Ehring, T., & Quack, D. (2010). Emotion regulation difficulties in trauma survivors: The role of trauma type and PTSD symptom severity. *Behavior Therapy*, *41*(4), 587–598. 10.1016/j.beth.2010.04.004 21035621

[CIT0016] European Monitoring Centre for Drugs and Drug Addiction (EMCDDA). (2018). *Drug consumption rooms: An overview of provision and evidence*.

[CIT0017] Fairbairn, N., Small, W., Shannon, K., Wood, E., & Kerr, T. (2008). Seeking refuge from violence in street-based drug scenes: Women's experiences in North America's first supervised injection facility. *Social Science & Medicine*, *67*(5), 817–823. 10.1016/j.socscimed.2008.05.012 18562065

[CIT0018] Garami, J., Valikhani, A., Parkes, D., Haber, P., Mahlberg, J., Misiak, B., Frydecka, D., & Moustafa, A. A. (2019). Examining perceived stress, childhood trauma and interpersonal trauma in individuals with drug addiction. *Psychological Reports*, *122*(2), 433–450. 10.1177/0033294118764918 29569991

[CIT0019] Goodhew, M., Salmon, A. M., Marel, C., Mills, K. L., & Jauncey, M. (2016). Mental health among clients of the Sydney Medically Supervised Injecting Centre (MSIC). *Harm Reduction Journal*, *13*(1), 1–5. 10.1186/s12954-016-0117-y 27733167 PMC5062820

[CIT0020] Gray, M. J., Litz, B. T., Hsu, J. L., & Lombardo, T. W. (2004). Psychometric properties of the life events checklist. *Assessment*, *11*(4), 330–341. 10.1177/1073191104269954 15486169

[CIT0021] Hall, N. Y., Le, L., Abimanyi-Ochom, J., Teesson, M., & Mihalopoulos, C. (2023). Identifying the most common barriers to opioid agonist treatment in an Australian setting. *Australian Journal of Primary Health*, *29*(5), 445–454. 10.1071/PY22269 36934460

[CIT0022] Harris, P. A., Taylor, R., Minor, B. L., Elliott, V., Fernandez, M., O'Neal, L., McLeod, L., Delacqua, G., Delacqua, F., Kirby, J., & Duda, S. N. (2019). The REDCap consortium: Building an international community of software platform partners. *Journal of Biomedical Informatics*, *95*, 103208. 10.1016/j.jbi.2019.103208 31078660 PMC7254481

[CIT0023] Harris, P. A., Taylor, R., Thielke, R., Payne, J., Gonzalez, N., & Conde, J. G. (2009). Research electronic data capture (REDCap)—A metadata-driven methodology and workflow process for providing translational research informatics support. *Journal of Biomedical Informatics*, *42*(2), 377–381. 10.1016/j.jbi.2008.08.010 18929686 PMC2700030

[CIT0024] Harvey, L. R., Hopkins, R., Truscott, M., Marel, C., Slade, T., & Mills, K. L. (2023). A retrospective chart review of trauma-related documentation in an Australian substance use treatment service. *Drug and Alcohol Review*, *42*(2), 373–383. 10.1111/dar.13575 36377196 PMC10947072

[CIT0025] IBM Statistics. (2013). *IBM corp. Released 2013. IBM SPSS statistics for windows, version 22.0*. IBM Corp. Google Search.

[CIT0026] Ivsins, A., Warnock, A., Small, W., Strike, C., Kerr, T., & Bardwell, G. (2023). A scoping review of qualitative research on barriers and facilitators to the use of supervised consumption services. *International Journal of Drug Policy*, *111*, 103910. 10.1016/j.drugpo.2022.103910 36436364

[CIT0027] Kantor, V., Knefel, M., & Lueger-Schuster, B. (2017). Perceived barriers and facilitators of mental health service utilization in adult trauma survivors: A systematic review. *Clinical Psychology Review*, *52*, 52–68. 10.1016/j.cpr.2016.12.001 28013081

[CIT0028] Kennedy, M. C., Hayashi, K., Milloy, M.-J., Boyd, J., Wood, E., & Kerr, T. (2020). Supervised injection facility use and exposure to violence among a cohort of people who inject drugs: A gender-based analysis. *International Journal of Drug Policy*, *78*, 102692. 10.1016/j.drugpo.2020.102692 32200269 PMC7302974

[CIT0029] Kennedy, M. C., Hayashi, K., Milloy, M.-J., Wood, E., & Kerr, T. (2019). Supervised injection facility use and all-cause mortality among people who inject drugs in Vancouver, Canada: A cohort study. *PLoS Medicine*, *16*(11), e1002964. 10.1371/journal.pmed.1002964 31770391 PMC6879115

[CIT0030] Kerman, N., Manoni-Millar, S., Cormier, L., Cahill, T., & Sylvestre, J. (2020). “It’s not just injecting drugs”: Supervised consumption sites and the social determinants of health. *Drug and Alcohol Dependence*, *213*, 108078. 10.1016/j.drugalcdep.2020.108078 32485658

[CIT0031] Kimber, J., Mattick, R. P., Kaldor, J., Van Beek, I., Gilmour, S., & Rance, J. A. (2008). Process and predictors of drug treatment referral and referral uptake at the Sydney Medically Supervised Injecting Centre. *Drug and Alcohol Review*, *27*(6), 602–612. 10.1080/09595230801995668 19378444

[CIT0032] Larance, B., Lintzeris, N., Bruno, R., Peacock, A., Cama, E., Ali, R., Kihas, I., Hordern, A., White, N., & Degenhardt, L. (2015). The characteristics of a cohort who tamper with prescribed and diverted opioid medications. *Journal of Substance Abuse Treatment*, *58*, 51–61. 10.1016/j.jsat.2015.06.001 26286818

[CIT0033] López-Castro, T., Sohler, N., Riback, L., Bravo, G., Ohlendorf, E., Ghiroli, M., & Fox, A. D. (2024). Posttraumatic stress disorder in people who use drugs: Syringe services program utilization, treatment need, and preferences for onsite mental health care. *Harm Reduction Journal*, *21*(1), 108. 10.1186/s12954-024-01019-5 38824597 PMC11143655

[CIT0034] Manning, V., Garfield, J. B. B., Lam, T., Allsop, S., Berends, L., Best, D., Buykx, P., Room, R., & Lubman, D. I. (2019). Improved quality of life following addiction treatment is associated with reductions in substance use. *Journal of Clinical Medicine*, *8*(9), 1407. 10.3390/jcm8091407 31500211 PMC6780566

[CIT0035] Palic, S., Zerach, G., Shevlin, M., Zeligman, Z., Elklit, A., & Solomon, Z. (2016). Evidence of complex posttraumatic stress disorder (CPTSD) across populations with prolonged trauma of varying interpersonal intensity and ages of exposure. *Psychiatry Research*, *246*, 692–699. 10.1016/j.psychres.2016.10.062 27839826

[CIT0036] Panayi, P., Berry, K., Sellwood, W., Campodonico, C., Bentall, R. P., & Varese, F. (2022). The role and clinical correlates of complex post-traumatic stress disorder in people with psychosis. *Frontiers in Psychology*, *13*, 791996. 10.3389/fpsyg.2022.791996 35369153 PMC8967251

[CIT0037] Peirce, J. M., Kolodner, K., Brooner, R. K., & Kidorf, M. S. (2012). Traumatic event re-exposure in injecting drug users. *Journal of Urban Health*, *89*(1), 117–128. 10.1007/s11524-011-9619-9 21989498 PMC3284586

[CIT0038] Rance, J., & Fraser, S. (2011). Accidental intimacy: Transformative emotion and the Sydney Medically Supervised Injecting Centre. *Contemporary Drug Problems*, *38*(1), 121–145. 10.1177/009145091103800106

[CIT0039] Randhawa, G., Azarbar, A., Dong, H., Milloy, M. J., Kerr, T., & Hayashi, K. (2018). Childhood trauma and the inability to access hospital care among people who inject drugs. *Journal of Traumatic Stress*, *31*(3), 383–390. 10.1002/jts.22286 29924415 PMC6026062

[CIT0040] Rapp, R. C., Xu, J., Carr, C. A., Lane, D. T., Wang, J., & Carlson, R. (2006). Treatment barriers identified by substance abusers assessed at a centralized intake unit. *Journal of Substance Abuse Treatment*, *30*(3), 227–235. 10.1016/j.jsat.2006.01.002 16616167 PMC1986793

[CIT0041] Rasmussen, A., Romero, S., Leon, M., Verkuilen, J., Morales, P., Martinez-Maganalles, S., & García-Sosa, I. (2020). Measuring trauma exposure: Count versus variety of potentially traumatic events in a binational sample. *Journal of Traumatic Stress*, *33*(6), 973–983. 10.1002/jts.22563 32598570

[CIT0042] Reed, G. M., First, M. B., Billieux, J., Cloitre, M., Briken, P., Achab, S., Brewin, C. R., King, D. L., Kraus, S. W., & Bryant, R. A. (2022). Emerging experience with selected new categories in the ICD-11: Complex PTSD, prolonged grief disorder, gaming disorder, and compulsive sexual behaviour disorder. *World Psychiatry*, *21*(2), 189–213. 10.1002/wps.20960 35524599 PMC9077619

[CIT0043] Rickard, G., & Hart, B. (2022). Survival, safety and belonging: An ethnographic study of experiences and perceptions of people who inject drugs accessing a supervised injecting Centre. *Australian Journal of Social Issues*, *57*(4), 829–846. 10.1002/ajs4.230

[CIT0044] Ryan, J. (2023). *Review of the medically supervised injecting room final report: Key findings and recommendations*. State of Victoria, Department of Health: 1 Treasury Place.

[CIT0045] Salter, M., Conroy, E., Dragiewicz, M., Burke, J., Ussher, J., Middleton, W., Vilenica, S., Martin Monzon, B., & Noack-Lundberg, K. (2020). *“A deep wound under my heart”: Constructions of complex trauma and implications for women’s wellbeing and safety from violence* (Research report, 12/2020). ANROWS.

[CIT0046] Santo, T., Campbell, G., Gisev, N., Martino-Burke, D., Wilson, J., Colledge-Frisby, S., Clark, B., Tran, L. T., & Degenhardt, L. (2022). Prevalence of mental disorders among people with opioid use disorder: A systematic review and meta-analysis. *Drug and Alcohol Dependence*, *238*, 109551. 10.1016/j.drugalcdep.2022.109551 35797876

[CIT0047] Scheim, A. I., Sniderman, R., Wang, R., Bouck, Z., McLean, E., Mason, K., Bardwell, G., Mitra, S., Greenwald, Z. R., Thavorn, K., Garber, G., Baral, S. D., Rourke, S. B., & Werb, D. (2021). The Ontario integrated supervised injection services cohort study of people who inject drugs in Toronto, Canada (OiSIS-Toronto): cohort profile. *Journal of Urban Health*, *98*(4), 538–550. 10.1007/s11524-021-00547-w 34181179 PMC8237772

[CIT0048] Small, W., Moore, D., Shoveller, J., Wood, E., & Kerr, T. (2012). Perceptions of risk and safety within injection settings: Injection drug users’ reasons for attending a supervised injecting facility in Vancouver, Canada. *Health, Risk & Society*, *14*(4), 307–324. 10.1080/13698575.2012.680950

[CIT0049] Spector, A. L., Quinn, K. G., Young, S. A., O'Brien, M., deRoon-Cassini, T. A., & Dickson-Gomez, J. (2021). A qualitative examination of substance use disorder treatment-seeking among women with opioid use disorders: The role of syndemics and structural violence. *SSM-Qualitative Research in Health*, *1*, 100014. 10.1016/j.ssmqr.2021.100014

[CIT0050] Substance Abuse and Mental Health Services Administration (SAMHSA). (2014). *SAMHSA's concept of trauma and guidance for a trauma-informed approach*. US Department of Health and Human Services.

[CIT0051] Treloar, C., Rance, J., Yates, K., & Mao, L. (2016). Trust and people who inject drugs: The perspectives of clients and staff of Needle Syringe Programs. *International Journal of Drug Policy*, *27*, 138–145. 10.1016/j.drugpo.2015.08.018 26394538

[CIT0052] Urbanik, M.-M., Maier, K., & Greene, C. (2022). A qualitative comparison of how people who use drugs’ perceptions and experiences of policing affect supervised consumption services access in two cities. *International Journal of Drug Policy*, *104*, 103671. 10.1016/j.drugpo.2022.103671 35413583

[CIT0053] Van Den Boom, W., del Mar Quiroga, M., Fetene, D. M., Agius, P. A., Higgs, P. G., Maher, L., Hickman, M., Stoové, M. A., & Dietze, P. M. (2021). The Melbourne safe injecting room attracted people most in need of its service. *American Journal of Preventive Medicine*, *61*(2), 217–224. 10.1016/j.amepre.2021.02.018 34011443

[CIT0054] Weis, C. N., Webb, E. K., Stevens, S. K., Larson, C. L., & deRoon-Cassini, T. A. (2022). Scoring the life events checklist: Comparison of three scoring methods. *Psychological Trauma: Theory, Research, Practice, and Policy*, *14*(4), 714–720. 10.1037/tra0001049 34166045 PMC8702580

[CIT0055] Wilker, S., Pfeiffer, A., Kolassa, S., Koslowski, D., Elbert, T., & Kolassa, I.-T. (2015). How to quantify exposure to traumatic stress? Reliability and predictive validity of measures for cumulative trauma exposure in a post-conflict population. *European Journal of Psychotraumatology*, *6*(1), 28306. 10.3402/ejpt.v6.28306 26589255 PMC4654773

[CIT0056] Williamson, V., Pearson, E. J., Shevlin, M., Karatzias, T., Macmanus, D., & Murphy, D. (2021). Experiences of veterans with ICD-11 complex PTSD in engaging with services. *Journal of Loss and Trauma*, *26*(2), 166–178. 10.1080/15325024.2020.1749784

[CIT0057] Wood, E., Tyndall, M. W., Li, K., Lloyd-Smith, E., Small, W., Montaner, J. S. G., & Kerr, T. (2005). Do supervised injecting facilities attract higher-risk injection drug users? *American Journal of Preventive Medicine*, *29*(2), 126–130. 10.1016/j.amepre.2005.04.011 16005809

